# Modulation of Telomere Length by Mediterranean Diet, Caloric Restriction, and Exercise: Results from PREDIMED-Plus Study

**DOI:** 10.3390/antiox10101596

**Published:** 2021-10-12

**Authors:** María Fernández de la Puente, Pablo Hernández-Alonso, Silvia Canudas, Amelia Marti, Montserrat Fitó, Cristina Razquin, Jordi Salas-Salvadó

**Affiliations:** 1 Universitat Rovira i Virgili, Departament de Bioquímica i Biotecnologia, Unitat de Nutrició Humana, 43201 Reus, Spain; maria.fernandezdelapuente@urv.cat (M.F.d.l.P.); pablo.hernandez@alumni.urv.cat (P.H.-A.); 2 Institut d’Investigació Sanitària Pere Virgili (IISPV), Hospital Universitari San Joan de Reus, 43204 Reus, Spain; 3 Institute of Biomedical Research of Malaga (IBIMA), Virgen de la Victoria University Hospital, 29010 Malaga, Spain; 4 Physiopathology of Obesity and Nutrition Networking Biomedical Research Center (CIBEROBN), Carlos III Health Institute, 28029 Madrid, Spain; amarti@unav.es (A.M.); mfito@imim.es (M.F.); crazquin@unav.es (C.R.); 5 Department of Nutrition, Food Sciences and Physiology, University of Navarra, 31008 Pamplona, Spain; 6 Instituto de Investigación Sanitaria de Navarra (IdiSNA), University of Navarra, 31008 Pamplona, Spain; 7 Cardiovascular Risk and Nutrition Research Group (CARIN), Hospital del Mar Research Institute (IMIM), 08003 Barcelona, Spain; 8 Department of Preventive Medicine and Public Health, Instituto de Investigación Sanitaria de Navarra (IdiSNA), University of Navarra, 31008 Pamplona, Spain

**Keywords:** Mediterranean diet, telomere length, 8-hydroxydeoxyguanosine (8-OHdG)

## Abstract

Telomere length (TL) has been associated with aging and is determined by lifestyle. However, the mechanisms by which a dietary pattern such as the Mediterranean diet (MedDiet) affects TL homeostasis are still unknown. Our aim was to analyse the effect of an energy-restricted MedDiet with physical activity promotion (intervention group) versus an unrestricted-caloric MedDiet with no weight-loss advice (control group) on TL and 8-hydroxydeoxyguanosine (8-OHdG) plasma levels. In total, 80 non-diabetic participants with metabolic syndrome were randomly selected from the PREDIMED (PREvención con DIeta MEDiterránea)-Plus-Reus study. TL was measured by a hybridisation method and 8-OHdG levels by ELISA at baseline and after one year of intervention. Linear mixed models (LMM)—raw and after adjusting for potential confounders—were used to examine the associations between TL or 8-OHdG plasma levels by intervention group and/or time. A total of 69 subjects with available DNA samples were included in the analyses. A significant β-coefficient was found for time towards increasing values through the year of follow-up for TL (unadjusted β of 0.740 (95% CI: 0.529 to 0.951), and multivariable model β of 0.700 (95% CI: 0.477 to 0.922)). No significant βs were found, neither for the intervention group nor for the interaction between the intervention group and time. Regarding 8-OHdG plasma levels, no significant βs were found for the intervention group, time, and its interaction. Our results suggest that MedDiet could have an important role in preventing telomere shortening, but calorie restriction and exercise promotion did not provide an additional advantage concerning telomere length after one year of MedDiet intervention.

## 1. Introduction

Aging is defined as an irreversible deterioration based on a physiological integrity decrease affecting most living systems. Several hallmarks are followed by this progressive loss of function such as telomere attrition, genomic damage, epigenetic changes, etc. [1,2]. Telomeres, the ends of our linear genomic DNA, are structures responsible for maintaining the stability of eukaryote chromosomes [3]. In mammals, the 5′-TTAGGG-3′ tandem repeated sequence that defines telomeres is highly conserved [4]. Following cell division, telomeres shorten to a critical level, triggering replicative senescence, which is a key factor of cellular aging [5]. Telomerase is the enzymatic ribonucleoprotein complex that adds ‘TTAGGG’ repeats, allowing telomere elongation [6]. The regulation of the telomerase activity has been shown to be essential to maintain a healthy overall status through the regulation of telomere lengthening [7].

In humans, it has been suggested that the rate of telomere shortening is both age- and tissue dependent [8]. Lifestyle factors (e.g., diet, physical activity, alcohol intake, and smoking) have been considered important determinants of telomere length maintenance. Adherence to a healthy lifestyle was positively associated with a lengthening of the leukocyte telomeres [9], whereas an unhealthy lifestyle with a negative impact on oxidative stress, inflammation, blood pressure, and insulin resistance, may have an accelerated telomere shortening effect related to chronic aging diseases [10]. 

During the last years, different dietary approaches have been suggested to have beneficial effects on telomere homeostasis since nutritional and behavioural strategies have been shown to influence telomere length (reviewed in [11,12]). Mediterranean diet has largely demonstrated cardiovascular benefits (reviewed in [13,14]) but also may have additional beneficial effects on telomere length. In fact, the available evidence derived from a systematic review of cross-sectional and prospective studies suggests that some antioxidant nutrients, the consumption of fruits, vegetables, and seeds (nuts, grains, and coffee), and Mediterranean diet (MedDiet) adherence are mainly associated with longer telomeres, suggesting a protective effect of some plant-based food compounds in the prevention of telomere shortening [15]. 

In a recent systematic review and meta-analysis of cross-sectional studies, higher MedDiet adherence was associated with longer telomeres [16]. However, no association was found between these two variables in the Helsinki Birth Cohort Study (HBCS), the only 10-year prospective cohort study conducted until now in a large Finnish middle-aged population [17]. 

To date, only one randomised controlled trial (RCT) has evaluated the effect of the MedDiet on telomere shortening after 5 y of follow-up, showing no effect of the MedDiet supplemented with extra virgin olive oil or nuts on telomere length changes when compared with a control group advised to follow a low-fat diet [18]. 

In order to delve deeper into the study of MedDiet as a key factor for the prevention of aging processes related to telomere length and/or 8-hydroxydeoxyguanosine (8-OHdG) plasma levels, we designed this study to evaluate the effect of an intensive lifestyle intervention in individuals with cardiometabolic risk, with weight-loss encouragement based on an energy-reduced MedDiet, compared with usual care, and participants were advised to follow a MedDiet without energy restriction on telomere length and 8-OHdG plasma levels in the framework of the PREDIMED (PREvención con DIeta MEDiterránea)-Plus study after one year of follow-up.

## 2. Materials and Methods

### 2.1. Study Design

This pilot study was conducted in the context of the PREDIMED-Plus trial, a 6-year parallel-group, multicentre RCT involving 6874 participants recruited in 23 Spanish recruiting centres. The PREDIMED-Plus protocol has been detailed elsewhere [19], and it was registered at the International Standard Randomised Controlled Trial (ISRCT; http://www.isrctn.com/ISRCTN89898870; accessed date: 11 October 2021) with number 89898870 (Registration date: 24 July 2014). However, the current report is aimed to evaluate the effect of the intensive lifestyle intervention on genomic DNA telomere length in comparison with usual care after 1-year follow-up. This analysis represents a nested sub-study performed in the local centre of Reus (PREDIMED-Plus-Reus). The local institutional review board approved the study protocol, and all participants provided written informed consent.

### 2.2. Study Subjects

In this sub-study conducted with participants of the PREDIMED-Plus Reus centre, a total of 80 non-diabetic participants with metabolic syndrome were randomly selected from those randomised to the control group (*n* = 40) and the intervention group (*n* = 40). Due to the lack of baseline or 1-year follow-up samples from 2 participants of the control group and 9 of the intervention group, a total of 69 subjects were included in the analysis. Participants allocated to the intervention group followed an energy-restricted MedDiet (erMedDiet) and physical activity promotion with specific weight loss objectives and individualised behavioural support. Participants in the control group were aimed to maintain an unrestricted caloric MedDiet with no advice on weight loss strategies.

Eligible participants were women aged 60–75 years and men aged 55–75 years with no documented history of cardiovascular disease at enrolment, who were overweight or obese (BMI 27–40 kg/m^2^), and who had at least three components of the metabolic syndrome. The main exclusion criteria were (1) active malignant cancer or history of malignancy within the last 5 years; (2) inability to follow the recommended diet or to perform physical activities; (3) history of surgical procedures for weight loss or intention to undergo bariatric surgery; (4) history of bowel resection or inflammatory bowel disease; (5) obesity of unknown endocrine origin; (6) food allergy to any component of the MedDiet; (7) immunodeficiency or HIV-positive status; (8) cirrhosis or liver failure; (9) serious psychiatric disorders; (10) severe co-morbidity condition; (11) alcohol or drug abuse; (12) history of major organ transplantation; (13) type 2 diabetes; (14) therapy with immunosuppressive drugs, cytotoxic agents, treatment with systemic corticosteroids, use of weight loss medication, etc. [20].

### 2.3. Blood Samples and DNA Extraction

Blood samples were collected after an overnight fast (10 h) at baseline and after a 1-year intervention period. Aliquots were stored at −80 °C, and measurements of the levels of serum total cholesterol, high-density lipoprotein cholesterol (HDL-cholesterol), and triglycerides were conducted using routine enzymatic methods. Low-density lipoprotein cholesterol (LDL-cholesterol) concentration was calculated by the Friedwald formula. Peripheral blood mononuclear cells (PBMCs) were isolated from whole blood by Ficoll-Hypaque density gradient centrifugation within 6 h of drawing blood. Genomic DNA was extracted from PBMCs by PureLink^TM^ Genomic DNA Mini Kit (Invitrogen, Madrid, Spain). 

### 2.4. Telomere Length Determination

Telomere length was measured with the use of a QuantiGene Plex DNA assay method (Termo Fisher Scientific, Madrid, Spain) using custom-designed probes to measure the abundance of the telomere repeat sequence [21]. The fluorescence signal after hybridisation was read on a Luminex flow cytometer (Termo Fisher Scientific, Madrid, Spain). Signal was reported as median fluorescence intensity (MFI), and it is proportional to the number of target DNA molecules present in the sample. ALK was used for reference single gen [21]. The assay was carried out in a 96-well plate with a fixed amount of 50 ng of genomic DNA, and all samples were measured in duplicate, and repeated measures samples were run in the same batch. Subjects with samples having a coefficient of variation higher than 5% were fully reanalysed.

### 2.5. Measurement of 8-Hydroxydeoxyguanosine Plasma Levels 

Levels of 8-OHdG were analysed at baseline and after a 1-year follow-up period in plasma samples. A volume of 20 µL of plasma sample was used for the quantitative measurement of 8-OHdG with the use of an OxiSelect Oxidative DNA damage ELISA Kit (Cell Biolabs, Inc., San Diego, CA, USA, EEUU). according to the manufacturer’s instructions. An ELISA conventional plate reader (Fluoroskan Ascent; Thermo Fisher Scientific, Madrid, Spain) was used to monitor the colorimetric substrate at a wavelength of 450 nm. All samples were measured in duplicate and those with a coefficient of variation greater than 2.5% were reanalysed. Samples from the same subject were included in the same plate.

### 2.6. Assessment of Covariates 

Body weight, waist circumference, and height were measured twice at baseline and after one year of follow-up, with the subjects wearing no shoes and light clothes. Body mass index (BMI) was calculated at the beginning and at the end of the 1-year period as weight (kg) divided by the square of height (m^2^). Blood pressure was measured using a validated semiautomatic oscillometer (Omron HEM-705CP, Hoofddorp, The Netherlands) in the non-dominant arm and after 5 min of rest in-between measurements. Except for blood pressure determined in triplicate, the remaining anthropometric variables were determined in duplicate, and the mean of these measurements was used. Dietitians used a validated food frequency questionnaire to estimate dietary consumption [22] and a 17-point validated tool [23] to calculate the energy-restricted MedDiet adherence score (erMedDiet score). Leisure-time physical activity was assessed using the validated REGICOR questionnaire [24] (including questions to collect information on the type of activity, frequency (number of days), and duration (min/day)).

### 2.7. Statistical Analyses 

Descriptive data of participants at baseline and differences during the intervention periods are shown as means (SD) for continuous variables, and number (%) for categorical variables. Descriptive analysis was conducted by using the chi-squared test for categorical variables and ANOVA for continuous variables. Telomere length and 8-OHdG plasma levels data were first transformed by applying log2. 

We used linear mixed models with a random intercept for each participant and an unstructured correlation matrix to examine the associations of changes in telomere length and 8-OHdG plasma levels according to the intervention group, time, and its interaction (intervention group x time). In all models, we used robust variance estimators to account for intra-cluster correlations, taking into account the members of the same household who were randomised together. We adjusted for the following fixed covariates: sex (men/women), age (continuous), education (primary/secondary/university), and smoking (never/former/current); and time-varying covariates: BMI (continuous), total energy intake (continuous), 17-point erMedDiet score (continuous), physical activity (continuous), and alcohol intake (continuous). We examined potential interactions with age, sex, and BMI by including interaction terms and comparing models using ANOVA. All models are presented as crude (unadjusted) and fully adjusted. All analyses were performed with R version 6.3.0. (The R Foundation, Vienna, Austria) and packages “tableone” [25], “lme4” [26] and “lmerTest” [27]. Statistical significance was set at *p* value <0.05.

## 3. Results

### 3.1. Baseline Characteristics of Control and Intervention Group Participants

This pilot sub-study was conducted in a total of 69 PREDIMED-Plus participants. Baseline participants’ characteristics according to the intervention group are presented in Table 1. No significant baseline differences between intervention groups were found for sex, age, weight, BMI, number of metabolic syndrome components, obesity, or prediabetes status at baseline.

### 3.2. Food Consumption 

Baseline and 1-year changes in food consumption are shown in Table 2. Participants in the intervention group experienced a significant decrease in the total energy intake (*p* = 0.013), while those in the control group experienced a non-significant decrease. During the intervention, subjects in the control group increased the intake of monounsaturated and polyunsaturated fatty acids (*p* = 0.001 and *p* = 0.02, respectively) and decreased the intake of saturated fatty acids (*p* = 0.013), whereas participants in the intervention group increased the levels of monounsaturated and polyunsaturated fatty acids, proteins and fibre (*p* ≤ 0.001, *p* ≤ 0.001, *p* ≤ 0.001 and *p* ≤ 0.001, respectively) and reduced the intake of carbohydrates (*p* ≤ 0.001). Participants in the intervention group increased the consumption of vegetables, legumes, nuts, and whole-grain cereals (*p* = 0.003, *p* ≤ 0.001, and *p* ≤ 0.001, *p* = 0.001, respectively) and decreased the consumption of sugary drinks and refined cereals (*p* = 0.039 and *p* ≤ 0.001, respectively), while in the control group, participants increased the consumption of virgin olive oil and nuts (*p* = 0.03 and *p* ≤ 0.001, respectively). Neither control group nor intervention group significantly changed the consumption of fruits (*p* = 0.713 and *p* = 0.768, respectively). 

A higher decrease in the consumption of total energy and carbohydrate (*p* = 0.015 and *p* ≤ 0.001, respectively) and a higher increase in monounsaturated (*p* = 0.005) and polyunsaturated fatty acids (*p* = 0.025), protein intake (*p* ≤ 0.001), and fibre (*p* = 0.041) were shown in participants of the intervention group, compared with the control group. No significant differences in changes between groups were found in the case of saturated fatty acids intake. Significant differences in changes between groups were found in the consumption of legumes (*p* ≤ 0.001), refined cereals (*p* ≤ 0.001),) and red wine (*p* = 0.01). Compared with participants in the control group, those in the intervention group showed a higher increase in the consumption of legumes. A decrease in the consumption of refined cereals and an increase in red wine intake in the intervention group, compared with the control group, was observed. No significant differences in changes between groups were observed in relation to the consumption of vegetables, fruits, dairy products, meat, sea and seafood, virgin olive oil, nuts, sugar-sweetened or sugar-free drinks, and whole-grain cereals.

### 3.3. Anthropometric Measurements and Biochemical Parameters 

Table 3 shows baseline and 1-year changes in anthropometric measurements, biochemical parameters, telomere length, and 8-OHdG plasma levels. After 1-year of follow-up, a significant reduction in BMI and waist circumference was observed in participants of the intervention group (*p* = 0.033 and *p* = 0.001, respectively), while participants in the control group showed no variation in these anthropometric measurements. Subjects in both intervention groups showed a decrease in triglycerides levels; however, this decrease was only significant in the case of the intervention group (*p* = 0.026). No significant changes in relation to the levels of total cholesterol, LDL-cholesterol, HDL-cholesterol, plasma glucose, HbA1c, or 8-OHdG plasma levels were observed, neither in participants in the control group nor in those in the intervention group. Participants in the intervention group experienced a significant increase in the total leisure-time physical activity from baseline (*p* = 0.02), while those in the control group experienced a non-significant decrease. Subjects in both the control and intervention groups experienced a significant increase in telomere length (*p* ≤ 0.001 and *p* = 0.001, respectively).

Significant differences in changes between groups were observed in different parameters. Compared with the control group, a higher decrease in total body weight (*p* ≤ 0.001), BMI (*p* ≤ 0.001) and waist circumference (*p* ≤ 0.001), and a higher increase in HDL-cholesterol (*p* = 0.001) concentrations, and leisure-time physical activity (*p* ≤ 0.001) was shown in those participants of the intervention group. No significant differences in changes between groups were found in relation to total and LDL-cholesterol, triglycerides, glucose, HbA1c, telomere length, or 8-OHdG plasma levels. In addition, both groups showed a greater improvement in the 17-point erMedDiet score, which was also significantly different between the two intervention groups.

### 3.4. Telomere Length and 8-OHdG Plasma Levels 

Changes in telomere length and 8-OHdG plasma levels by intervention group, time, and by the interaction between intervention group and time (effect of the intervention) are shown in Table 4. In the case of telomere length, the effect of the intervention was not statistically significant, in both the unadjusted (β coefficient of -0.142 (95% CI: −0.453 to 0.169) and multivariable-adjusted (β of −0.310 (95% CI: −0.650 to 0.025) models. Moreover, no significant β coefficient was found, neither for the intervention group (0.014 (95% CI: −0.206 to 0.234) in the unadjusted model nor the multivariable model (0.070 (95% CI: −0.150 to 0.289). Interestingly, a significant β was found for time towards increasing values through the follow-up. In the multivariable-adjusted model, the β coefficient was 0.700 (95% CI: 0.477 to 0.922). No significant changes in 8-OHdG plasma levels between intervention groups were observed. 

## 4. Discussion

In this PREDIMED-Plus sub-study, we observed greater adherence to the specific recommendations (higher increase in the MedDiet adherence and leisure-time physical activity), and greater metabolic benefits (higher weight loss and increase in HDL concentrations) in participants in the intervention group following an energy-restricted MedDiet and physical activity recommendations, than in those in the control group in which we advised to follow an unrestricted caloric MedDiet with no advice on weight loss. Despite these differences between groups, we found a favourable change in telomere length in both groups during the follow-up without significant differences observed between intervention groups. In addition, no significant changes in oxidative 8-OHdG plasma levels were shown throughout the follow-up in any of the intervention groups.

The lack of differences between intervention groups in relation to telomere length can be explained because both interventions have produced beneficial effects on MedDiet adherence. In fact, not enough significant differences between intervention groups have been established in terms of metabolic benefits, and it has been reflected in the absence of differences in LDL-cholesterol, blood pressure, glucose concentrations, glycated haemoglobin, and 8-OHdG plasma levels between groups. The increase in MedDiet adherence in both intervention groups could be responsible for this favourable change in telomere length within groups observed in our trial, although the mechanisms implicated are largely unknown. 

Several studies have shown how healthy dietary patterns may potentially impact telomeres maintenance. Our results are in line with a recent systematic review and meta-analysis of cross-sectional studies pooling data from different cohorts and performed by our group, in which we demonstrated that an increase in MedDiet adherence is associated with longer telomeres [16]. Although in a unique prospective cohort study analysing the association between MedDiet adherence and telomere lengthening, no significant association was found for the entire population including adult men and women, this association was significant in the case of women for whom MedDiet adherence was associated with faster telomere shortening after 10 y of follow-up, even though the effect estimate was small and thus clinically insignificant [17].

The results of our study differ from those reported in the PREDIMED-Navarra study evaluating the effect of a MedDiet intervention supplemented with either virgin olive oil or nuts, compared with a low-fat diet, on the risk of telomere shortening after 5 years of follow-up in a population of high cardiovascular disease [18]. Notably, in the PREDIMED-Navarra, subjects allocated to the MedDiet supplemented with extra virgin oil group showed no beneficial effect in telomere erosion in comparison with the control low-fat group, whereas a detrimental effect in telomere shortening was observed in the MedDiet supplemented with the nuts group.

These contradictory results can be explained by differences in the population studied, the interventions used, but also because in the PREDIMED-Navarra study, telomere length changes were measured by qPCR and here, by hybridisation using a flow cytometer. 

In a recent systematic review, it has been reported that high consumption of healthy foods typical in the traditional MedDiet pattern (vegetables, fruits, nuts, wine, and coffee, foods rich in antioxidants and other phytochemicals) was consistently associated with telomere lengthening, while high consumption of meat and processed meat and sweetened beverages may have the opposite effect [15]. In fact, in our study, we observed a tendency toward an increase in the consumption of different MedDiet foods such as vegetables, fruits, legumes, nuts, virgin olive oil, and whole-grain cereals, and a decrease in the consumption of meat and sweetened beverages in both intervention groups. While these changes were significant in some cases in the intervention group or in the control group, in the case of nuts, this increase was significant in both groups. This increase in nuts consumption can partly explain our results, as these results are in line with our previous short-term RCT showing that the consumption of 57 g/day of pistachios for 4 months had potential effects in the prevention of telomere shortening in prediabetic subjects [28].

The mechanisms by which traditional Mediterranean food may protect from telomere shortening are probably multiple and largely unknown. Some plant-based key foods of the MedDiet are especially rich in antioxidants and anti-inflammatory compounds and thus have been implicated in telomere maintenance by different mechanisms (reviewed by [29]). In fact, the consumption of these plant-based foods has consistently demonstrated beneficial effects on several anti-inflammatory markers and in reducing oxidative stress and cardiovascular risk factors (reviewed by [30]).

García-Calzón et al. demonstrated in children and adolescents that higher dietary total antioxidant capacity of the diet and lower refined white bread consumption (both typical of the MedDiet) were associated with longer telomeres [31]. High adherence to MedDiet was also associated with longer leukocyte telomeres, whereas a negative correlation was found between telomere length and inflammation parameters in elderly subjects from Italy [32]. In the aforementioned RCT published by our group, when participants were following the pistachio-enriched diet, they showed a tendency to decrease levels of DNA oxidation in parallel to a lower decrease in telomere length [28].

In our study, we did not measure peripheral or tissue-specific biochemical markers of inflammation and oxidation in order to understand if these mechanisms are responsible for telomere lengthening observed in both intervention groups. However, we measured 8-OHdG levels in plasma as an indirect measure of oxidative stress in the body. MedDiet has been demonstrated to decrease levels of 8-OHdG in DNA from peripheral blood leukocytes [33]. Although in our study both intervention groups adhered to the MedDiet, we did not observe significant effects of the interventions in this biomarker for either of the intervention groups. However, we cannot discard that the PREDIMED-Plus MedDiet interventions could have had beneficial effects on inflammation and oxidative stress when measured by other biomarkers. 

Despite the fact that in the present study, we did not determine hormone or telomerase activity levels, other potential mechanisms by which the MedDiet and its components may have beneficial effects on telomere maintenance could be related to its capacity to regulate the integrity of the hypothalamic–pituitary–adrenal axis and the production of cortisol, leading to a reduction in stress levels (reviewed by [34]) through the downregulation of the influence of cortisol on telomerase activity [35] and regulating DNA methylation changes. However, the interactions between genetic, epigenetic, environmental, and lifestyle factors make the whole framework even more complex, and future studies evaluating these and other mechanisms are needed to support the hypothesis to better understand the effect of MedDiet on telomere maintenance. 

This study has some strengths and limitations that should be highlighted. Among the strengths, the absence of previous RCTs evaluating the association between telomere length and MedDiet defines this research as an original and novel study. We used a validated technique for telomere length measurement instead of the conventional single-plex qPCR, which allows higher specificity and precision [21]. Moreover, we employed LMMs that allowed the analysis of repeated measurements while adjusting for several time-varying confounders and for baseline values of telomere length and 8-OHdG plasma levels. 

On the other hand, the results should be understood in the context of some limitations. First, this is a pilot sub-study with only 69 subjects, and that may have limited the statistical power. Furthermore, one year of follow-up could be insufficient to evaluate the real effects of the diet on telomere length and 8-OHdG plasma levels. Second, as our study population was composed of individuals with overweight/obesity and metabolic syndrome and did not include subjects with type 2 diabetes, our results may not be applied to other populations such as healthy or to individuals—male and female—with type 2 diabetes. Third, in our study, we did not include the measurement of parameters such as inflammatory-related or telomerase activity to understand better our findings. Fourth, as we did not test a control group without following MedDiet, we could not assure that the results we observed in relation to telomere attrition were only the results of the effect of adhering to MedDiet. Fifth, we measured 8-OHdG levels in plasma as an indirect measure of oxidative damage in the body but not DNA oxidation of cells that may have been a more accurate measure of oxidative stress. Finally, we measured telomere length in PBMCs and not in other cell groups or tissues, although it has been studied that the use of PBMCs for this purpose is an appropriate approach [8].

## 5. Conclusions

In conclusion, our results suggest that MedDiet, an antioxidant-rich dietary pattern, could have an important role in preventing telomere shortening after 1 year of intervention, whereas calorie restriction and exercise promotion do not provide this additional advantage. However, further studies should evaluate the effect of MedDiet on oxidative stress and telomere length with a larger sample size and longer follow-up to further support the research benefits of healthy dietary patterns for promoting health and longevity.

## Figures and Tables

**Table 1:** Baseline characteristics of the subjects at randomisation before the start of the study.

Characteristics	Control Group (*n* = 38)	Intervention Group (*n* = 31)	*p*-Value
Age (years)	64.8 (5.1)	64.3 (5.1)	0.655
Male, *n* (%)	17 (44.7)	12 (38.7)	0.795
Weight (kg)	85.8 (13.1)	88.8 (14.2)	0.360
BMI (kg/m^2^)	32.1 (3.4)	33.1 (3.6)	0.254
Waist circumference (cm)	104.1 (10.4)	107.3 (8.4)	0.174
Number of metabolic syndrome components (%)			0.940
≤3 Components	23 (60.5)	18 (58.1)	
4 Components	10 (26.3)	8 (25.8)	
5 Components	5 (13.2)	5 (16.1)	
Obese status			0.333
Overweight (BMI ≥25 kg/m^2^) (%)	11 (28.9)	5 (16.1)	
Obesity (BMI ≥30 kg/m^2^) (%)	27 (71.1)	26 (83.9)	
Prediabetes * (%)	21 (55.3)	13 (41.9)	0.390
Dyslipidaemia (%)	28 (73.7)	15 (48.4)	0.056
High blood pressure (%)	29 (76.3)	25 (80.6)	0.888
Depression (%)	16 (42.1)	7 (22.6)	0.146
Smokers (%)			0.407
Current	4 (10.5)	4 (12.9)	
Former	17 (44.7)	9 (29.0)	
Never	17 (44.7)	18 (58.1)	
Educational level (%)			0.404
Primary school	24 (63.2)	16 (51.6)	
High school or bachelor	8 (21.1)	12 (38.7)	
University	3 (7.9)	1 (3.2)	
Higher degree	3 (7.9)	2 (6.5)	
Medications use (%)			
Lipid-Lowering drugs			
Statin	18 (47.4)	10 (32.3)	0.408
Other lipid-lowering drugs	2 (5.3)	4 (12.9)	0.387
Hypotensive drugs			
Renin direct inhibitor	2 (5.3)	3 (9.7)	0.813
Angiotensin receptor blocker	8 (21.1)	8 (25.8)	0.660
Angiotensin converting enzyme inhibitor	14 (36.8)	12 (38.7)	0.742
Thiazide drugs ‡	18 (47.4)	13 (41.9)	0.750

Data are shown as means (standard deviation) or number (%); * prediabetes was defined as fasting plasma glucose of 100–125 mg/dL (5.6–6.9 mmol/L) or glycated haemoglobin (HbA1c) of 5.7–6.4% (39–47 mmol/mol). ‡ Thiazide drugs include thiazides and thiazide-like diuretics. *p* values for differences between groups by ANOVA or chi-squared test, as appropriate. Abbreviations: BMI, body mass index.

**Table 2:** Baseline and 1-year changes in energy and nutrient intake, total fat, and key food items by treatment groups.

	Control Group		Intervention Group		
	(*n* = 38)		(*n* = 31)		
	Baseline	Change	Baseline	Change	*p*-Value
Total energy intake (kcal/day)	2483.43 (602.53)	−72.44 (417.88)	2568.71 (543.51)	−334.94 (449.36) ¥	0.015
Protein (%)	15.54 (2.26)	0.27 (1.61)	15.78 (2.18)	2.14 (2.23) ¥	<0.001
Carbohydrate (%)	40.95 (4.98)	−1.37 (4.09)	41.40 (5.57)	−7.13 (6.12) ¥	<0.001
Fibre (g/d)	25.00 (9.48)	2.60 (7.08)	24.94 (6.45)	6.24 (7.37) ¥	0.041
Total fat (%)	40.02 (3.92)	1.58 (4.22)	39.87 (3.61)	5.16 (5.22) ¥	0.002
Monounsaturated fatty acids (%)	21.22 (3.10)	2.31 (3.44) ¥	21.06 (2.54)	4.82 (3.72) ¥	0.005
Polyunsaturated fatty acids (%)	6.31 (1.19)	0.88 (1.54) ¥	6.64 (1.20)	1.70 (1.38) ¥	0.025
Saturated fatty acids (%)	9.94 (1.58)	−0,85 (7.10) ¥	9.54 (1.55)	−0.31 (1.46)	0.166
Key food items					
Vegetables (g/d)	270.41 (105.07)	35.23 (104.99)	294.36 (86.35)	85.00 (137.87) ¥	0.093
Fruit (g/d)	341.49 (155.03)	11.18 (137.47)	355.50 (146.97)	9.84 (138.12)	0.968
Legumes (g/d)	19.38 (9.81)	0.45 (12.68)	17.65 (9.60)	11.21 (10.62) ¥	<0.001
Cereals (g/d)	160.01 (95.63)	9.03 (85.15)	180.09 (76.76)	−55.45 (84.81) ¥	0.003
Dairy products (g/d)	294.45 (188.68)	−47.26 (140.66)	244.13 (134.57)	6.85 (141.57)	0.118
Meat products (g/d)	154.46 (41.27)	−12.52 (36.33)	166.95 (59.29)	−21.20 (48.53)	0.399
Fish and seafood (g/d)	94.28 (43.08)	1.64 (51.53)	111.76 (44.65)	8.95 (45.28)	0.539
Nuts (g/d)	9.86 (10.91)	13.45 (12.98) ¥	14.06 (10.80)	18.13 (11.66) ¥	0.124
Red wine (ml/d)	59.16 (111.43)	−27.66 (61.46)	51.75 (76.98)	20.98 (91.30)	0.010
Virgin olive oil (g/d)	37.76 (19.48)	9.13 (14.85) ¥	41.98 (16.19)	6.08 (17.81)	0.440
Sugary drinks (ml/d)	31.80 (54.06)	−10.55 (67.35)	22.24 (41.27)	−16.83 (43.45) ¥	0.655
Sugar-free drinks (ml/d)	24.01 (89.13)	2.66 (65.32)	32.26 (179.61)	−30.91 (174.57)	0.277
Refined cereals (g/day)	118.15 (79.61)	−0.73 (63.42)	155.21 (94.56)	−97.18 (103.28) ¥	<0.001
Whole-grain cereals (g/day)	39.84 (93.80)	9.93 (81.45)	20.96 (41.31)	40.96 (47.70) ¥	0.065

Data are shown as means (SD) for continuous variables or number (%) for categorical variables for baseline characteristics. ¥ means statistical significance within a group. *p* values for differences between groups by ANOVA or chi-squared test, as appropriate.

**Table 3:** Baseline and 1-year changes in anthropometric measurements, biochemical parameters, telomere length, and 8-OHdG plasma levels by treatment groups.

	Control Group		Intervention Group		
	(*n* = 38)		(*n* = 31)		
	Baseline	Change	Baseline	Change	*p*-Value
Anthropometric measurements					
Body weight (kg)	85.76 (13.08)	−0.20 (2.18)	88.79 (14.15)	−5.51 (3.12)	<0.001
BMI (kg/m^2^)	32.11 (3.40)	−0.09 (0.81)	33.07 (3.58)	−2.05 (1.14) ¥	<0.001
Waist circumference (cm)	104.14 (10.39)	0.03 (3.24)	107.33 (8.44)	−7.94 (4.69) ¥	<0.001
Biochemical parameters					
Total cholesterol (mM)	217.63 (38.56)	−5.00 (40.81)	202.00 (34.93)	−5.68 (26.19)	0.937
LDL cholesterol (mM)	126.56 (33.16)	0.41 (41.12)	116.23 (20.41)	−1.23 (19.18)	0.853
HDL cholesterol (mmol/L)	51.82 (12.21)	−1.61 (6.10)	46.77 (13.14)	3.32 (5.47)	0.001
Triglycerides (mmol/L)	224.79 (167.78)	−20.76 (105.93)	208.10 (107.73)	−51.55 (76.07) ¥	0.179
Glucose (mmol/L)	101.08 (14.59)	−3.13 (12.96)	97.58 (12.65)	−5.29 (8.47)	0.428
HbA1c (%)	5.86 (0.50)	−0.07 (0.36)	5.73 (0.37)	−0.11 (0.26)	0.586
Leisure-time PA (METs.min/d)	385.02 (383.64)	−17.83 (307.89)	302.65 (262.30)	329.53 (403.68) ¥	<0.001
17-point erMedDiet score	8.00 (2.73)	2.53 (2.78) ¥	6.90 (2.55)	7.45 (2.72) ¥	<0.001
Telomere Length	3.14 (0.51)	0.65 (0.83) ¥	3.11 (0.80)	0.67 (0.80) ¥	0.922
8-OHdG plasma levels	2.84 (0.65)	0.01 (0.56)	2.95 (0.83)	0.12 (0.81)	0.528

Data are shown as means (SD) for continuous variables or number (%) for categorical variables for baseline characteristics. ¥ means statistical significance within a group. *p* values for differences between groups by ANOVA or chi-squared test, as appropriate. Telomere length and 8-OHdG plasma levels data were transformed applying log2. Abbreviations: 8-OHdG, 8-hydroxydeoxyguanosine; BMI, body mass index; erMedDiet, energy-restricted Mediterranean Diet; HbA1c, glycated haemoglobin; PA, physical activity.

**Table 4:** One-year changes in telomere length and 8-OHdG plasma levels according to the intervention group, time, and its interaction (effect of the intervention).

	β for Intervention Group ^1^	β for Time ^2^	β for Intervention Group ^1^ x Time ^2^
Telomere length			
Unadjusted model	0.014 (−0.206, 0.234)	0.740 (0.529, 0.951)	−0.142 (−0.453, 0.169)
Multivariable model	0.070 (−0.150, 0.289)	0.700 (0.477, 0.922)	−0.310 (−0.650, 0.025)
8-OHdG plasma levels			
Unadjusted model	0.122 (−0.212, 0.456)	−0.018 (−0.248, 0.213)	0.139 (−0.200, 0.478)
Multivariable model	0.041 (−0.172, 0.254)	−0.054 (−0.270, 0.163)	0.089 (−0.237, 0.415)

We used linear mixed models (with unstructured correlation matrix and robust variance estimators) to conduct these analyses. β coefficients (95% confidence intervals) are reported. ^1^ Intervention group versus control group; ^2^ visit at one-year follow-up versus at baseline. We adjusted for the following variables: intervention group, time, sex, age, BMI, education, total energy intake, 17-point erMedDiet score, physical activity, smoking, and alcohol intake. Additionally adjusted by baseline telomere length or 8-OHdG plasma levels values, as appropriate.

## Data Availability

The data are not publicly available outside of the core PREDIMED-Plus research group as neither participants’ consent forms nor ethics approval included permission for open access. However, the researchers will follow a controlled data-sharing collaboration model since in the informed consent, participants agreed with a controlled collaboration with other investigators for research related to the project’s aims. Therefore, investigators who are interested in this study can contact the PREDIMED Steering Committee by sending a request letter to jordi.salas@urv.cat. A data-sharing agreement indicating the characteristics of the collaboration and data management will be completed for the proposals that are approved by the Steering Committee.
